# Diatom diversity, distribution and ecology in Mediterranean ecosystems of Abrau Peninsula, north-western Caucasus

**DOI:** 10.3897/BDJ.10.e89405

**Published:** 2022-08-16

**Authors:** Alisa A. Neplyukhina, Ruslan A. Saifutdinov, Angelina A. Paskhina, Daniil I. Korobushkin

**Affiliations:** 1 Severtsov Institute of Ecology and Evolution RAS, Moscow, Russia Severtsov Institute of Ecology and Evolution RAS Moscow Russia; 2 Lomonosov Moscow State University, Moscow, Russia Lomonosov Moscow State University Moscow Russia

**Keywords:** Bacillariophyta, freshwater ecosystems, coastal ecosystems, new records, species check-list, Utrish State Nature Reserve

## Abstract

**Background:**

The North Caucasus is an extensive region with a multitude of landscapes and high biological diversity. Amongst various ecosystems, the xerophytic sub-Mediterranean forests of the Abrau Peninsula (Utrish State Nature Reserve) and its vicinity are unique but have been poorly studied. The diversity of diatoms in North Caucasian ecosystems have been studied partially and only little information is available about their presence and distribution on the Abrau Peninsula. Here, we present a comprehensive check-list of diatoms sampled during a July 2021 field campaign. Samples were collected in 67 sites, including 39 permanent streams, 21 temporal (puddles) and seven permanent waterbodies. Results of the current study contribute to improving the knowledge about diatoms in the north-western Caucasus and its sub-Mediterranean ecosystems in particular.

**New information:**

Here, we provide a detailed dataset that contains 215 freshwater and brackish diatom occurrences collected during a field campaign in July 2021. A total of 88 diatom (Bacillariophyta) taxa which belong to 12 orders, 25 families and 39 genera were collected. The genera with the highest number of occurrences per site were *Gomphonema* (26), *Nitzschia* (22), *Navicula* (20), *Cocconeis* (14), *Amphora* (14), *Achnanthidium* (14) and *Planothidium* (11). The genera with the highest number of infrageneric taxa were *Nitzschia* (8), *Navicula* (7), *Gomphonema* (6) and *Mastogloia* (5). *Naviculablazencicae*, known as the endemic of the Lake Prespa (Levkov 2007) is found from two sites in our study. Three specimens of the genus *Mastogloia* could not be assigned to a known species and may represent new diatom species. Distribution and ecology data are provided for each taxa. Occurrence data are given. Statistical analysis of diatom communities showed a significant dependence on habitat type and their ecological conditions.

## Introduction

Diatoms are a widely distributed group of algae whose representatives populate both aquatic (marine and freshwater) and terrestrial ecosystems, such as soils, mosses, wet walls and rocks (Round et al. 1990, Smol and Stoermer 2010) and play a key role in the nutrient cycle and energy flux (Benoiston et al. 2017). In seas and oceans, organic carbon produced by diatoms is consumed rapidly and serves as a base for marine food webs. In coastal waters, diatoms support most productive fisheries. In the open ocean, a relatively large proportion of diatom organic matter sinks rapidly from the surface, becoming food for deep-water organisms (Armbrust 2009). Soils and other terrestrial ecosystems have more severe effects for diatoms and differ from aquatic ecosystems in diatom species composition, although diatoms can be the dominant algal group at periods of the year with high soil moisture (Foets et al. 2020).

Diatoms are regularly used as biological indicators for the water quality environmental assessment (Patrick 1973, Reid et al. 1995, Kelly et al. 1998, Battarbee et al. 2002). The analysis of diatom communities and their biodiversity is a useful tool to secure an ecological and sustainable use of the water resources and the correct elaboration of guidelines for their preservation, in particular, in specially protected natural areas. Some recent studies have shown that natural springs in protected areas may act as biodiversity hotspots (Falasco and Bona 2011, Falasco et al. 2012).

Different ecological groups of Black Sea diatoms have been actively studied, especially from the perspective of water quality assessment (Petrov and Nevrova 2007, Ryabushko et al. 2017, Ryabushko et al. 2019, Ryabushko et al. 2021). Additionally, there is ongoing research on diatom diversity of specially protected natural sea areas of the Black Sea (Nevrova 2015, Ryabushko et al. 2018, Polyakova and Davidovich 2019, Ryabushko et al. 2021, Davidovich and Polyakova 2022). In a recent study of diatom communities in the water area surrounding Bolshoi Utrish (Anapa District, Russia), it was found that 77% of the biomass and 25% of the total phytoplankton abundance was composed of Bacillariophyta species (Yasakova and Kolesnikov 2021). Inland research of diatom communities have mainly been focused on Abrau Lake, the largest freshwater lake in the Abrau Peninsula (Kovaleva 2005, Kovaleva 2018). There are, however, a number of freshwater waterbodies and streams in the Abrau Peninsula and the nearby Black Sea coastal zone that are still understudied in terms of diatom diversity and distribution. We assume that some sampling locations (freshwater streams) on the territory of Utrish State Nature Reserve, especially without anthropogenic disturbance, potentially might be hidden hotspots of diatom biodiversity.

This study presents a taxonomical characterisation and occurrence dataset of the diatoms found in Mediterranean ecosystems of the Abrau Peninsula, north-western Caucasus, particularly in protected areas of Utrish State Nature Reserve. We aim to contribute to the current knowledge of diatom diversity and distribution in the freshwater and brackish inland water in the north-western Caucasus and its sub-Mediterranean ecosystems in particular.

## Project description

### Title

Diatom diversity, distribution and ecology in Mediterranean ecosystems of Abrau Peninsula, north-western Caucasus.

### Personnel

Samples were collected on 12-20 July 2021 on the Abrau Peninsula by Alisa Neplyukhina and Angelina Paskhina. Identifications were made by Alisa Neplyukhina. Statistical analyses were performed by Daniil Korobushkin and Ruslan Saifutdinov. The text was written by Alisa Neplyukhina, Daniil Korobushkin and Ruslan Saifutdinov.

### Study area description

The Abrau Peninsula is located between the city of Anapa and Abrau-Durso settlement in Novorossiysk District, Krasnodar Krai, Russia. Most of the Abrau Peninsula is under the protection of the Utrish State Nature Reserve (hereinafter referred to as "Utrish") and is not affected or disturbed by human activity, with the exception of the coastal zone and suburbs. The Abrau Peninsula has a humid subtropical (Cfa) and Mediterranean climate (Csa) according to the Köppen climate classification with cool rainy winters without stable snow cover and with hot dry summers (Chen and Chen 2013). The mean annual precipitation ranges from 480 mm (Anapa) to 788 mm (Novorossiysk), the mean July and February temperatures for both localities are 21℃ and 2℃, respectively (weatherbase, CantyMedia 2022).

The study area belongs to the Mediterranean ecoregion (Olson et al. 2001, Ogureeva et al. 2018) and is the only place in Russia covered by Mediterranean forests. The vegetation here forms three major belts (Bocharnikov et al. 2020, Seregin and Suslova 2007): 1. coastal slopes with sub-Mediterranean xerophytic forests and shrublands with pistachio (*Pistaciamutica*), juniper (*Juniperusexcelsa*, *J.oxycedrus*, *J.foetidissima*), oak (*Quercuspubescens*) and oriental hornbeam (*Carpinusorientalis*); 2. piedmont and low-mountain area with a combination of mesophilic and xerophilic forests and a predominance of two oak species (*Q.pubescens*, *Q.petrea*), oriental hornbeam and junipers; 3. low mountains with mesophilic deciduous forests with a domination of oak (*Q.petrea*), hornbeam (*Carpinuscaucasica*), lime (*Tiliabegoniifolia*), maple (*Acerlaetum*), ash (*Fraxinusexcelsior*) and beech (*Fagusorientalis*). A distinctive feature of the Utrish flora is unique pronounced Mediterranean core tertiary relict elements. It is inhabited by numerous rare, endemic species of flora and fauna.

Freshwater habitats are represented by permanent and temporary streams flowing to the Black Sea, as well as temporary small waterbodies (hereinafter referred as "puddles") scattered across the Abrau Peninsula. Brackish habitats are represented by small permanent lagoons located along the coastline.

## Sampling methods

### Study extent

**Figure 1.**
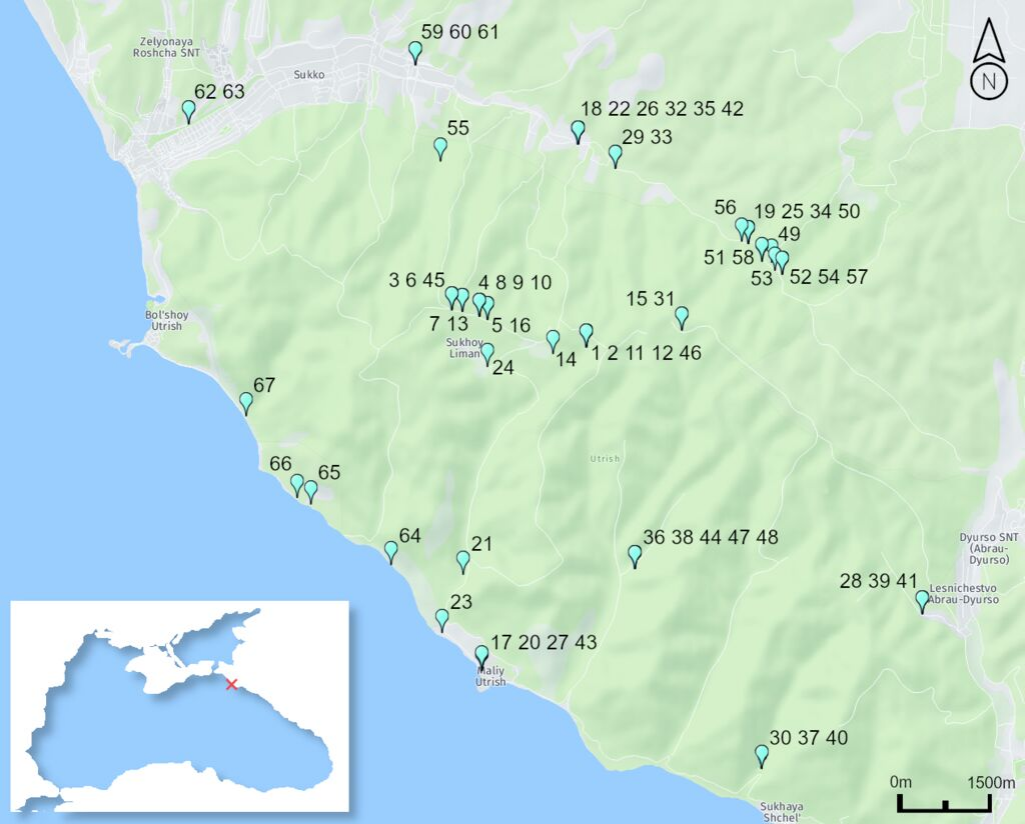
Study area and sampling sites location (Map source credits: https://wego.here.com).

Diatoms were collected from 67 sampling sites, including 39 permanent streams, 21 temporal waterbodies (puddles) and seven permanent waterbodies (lakes and lagoons) collected on the Abrau Peninsula, north-western Caucasus, Russia (Fig. 1, Table 1).

### Sampling description

Material for this research was collected in July 2021. Sampling was carried out after the annual peak of summer precipitation in June (CantyMedia 2022) and performed after a week of strong rains (CantyMedia 2022). This made it possible to collect material from both permanent and temporary waterbodies. Diatom samples were collected from 67 sites on the Abrau Peninsula (Table 1). The sampling sites differed in salinity from brackish to freshwater. Sample types include 36 rock scrap samples, 24 sediments samples, six soil samples and one moss squeeze sample. Diatom samples were collected in 50 ml plastic containers and immediately fixed with Lugol's solution (2 ml to 50 ml of sample) in order to keep other algae groups in their best condition for futher research (Sadchikov 2003). Material was cleaned from the organics in accordance with the hot peroxide method following Kelly et al. (2001). Light microscopical investigations were performed in bright-field optics using a Leica DM 750 microscope, equipped with a Leica ICC50 HD digital camera. Permanent slides were prepared with Naphrax®. For the scanning electron microscopy investigation, drops of cleaned material were air-dried on pieces of aluminium foil, mounted on brass stubs with double-sided carbon tape and coated with Au in a S150A Sputter Coater (Edwards, UK) ion coater. Scanning electron microscopic investigations were conducted using TESCAN MIRA 3 LMH (TESCAN, Czech Republic) in the Joint Usage Center «Instrumental methods in ecology» at the IEE RAS. All prepared LM slides and SEM stubs are stored in the collection of the Laboratory for Ecology of Aquatic Communities and Invasions, IEE RAS.

### Quality control

For diatom identification, a number of manuals were used (Lange-Bertalot 2001, Kulikovskiy et al. 2016, Cantonati et al. 2017). Valid diatom taxon names were verified according to Guiry and Guiry (2022). Data on diatom ecology are given according to Kulikovskiy et al. (2016), Cantonati et al. (2017) and Guiry and Guiry 2022 .

### Step description

The data have been published as a Darwin Core Archive (DwC-A), which is a standardised format for sharing biodiversity data as a set of one or more data tables. The core data table contains 215 occurrences (Neplyukhina et al. 2022).

**Statistical analysis**: Similarity between diatom communities of Abrau Peninsula was evaluated using hierarchical cluster analysis. Before analysis, the data were prepared via the dplyr 1.0.8. package (Wickham et al. 2018) into species x communities matrix with presence/absence data. Data on diatoms were pooled into communities according to their presence in the habitat type (stream, waterbody or puddle) and according to the sampling method (scrap, sediment, moss and soil). A detailed description of habitat type selection and sampling methods is given in Table 1. Distances between communities were calculated using a binary method and the Ward.D2 method was selected for the hierarchical clustering procedure. Additionally for each cluster, bootstrap probability value (BP) and approximately unbiased (AU) probability values (*p-values*) were calculated via multi-scale bootstrap on 10000 resamplings using the package pvclust 2.2-0 (Suzuki and Shimodaira 2006). To define our clusters, we used a significance level of p < 0.05, i.e. the AU value equal or higher than 95. The obtained dendrogram was customised with the dendextend 1.15.2 package (Galili 2015). The above analyses were performed in R 4.1.2 (R Core Team 2021) with R Studio interface (R studio Inc.). To analyse the correlation between the species richness of diatoms belonging to a particular ecological group and their presence in various habitats of the Abrau Peninsula, the principal component analysis (PCA) was applied. Sampled habitats (freshwater puddles, freshwater streams, freshwater waterbodies and brackish waterbodies in accordance with Table 1) and separability preferences (eutrophic, mesotrophic, oligotrophic, polluted water) were selected as active variables, while environment preferences (freshwater, brackish, marine) were chosen as additional (passive) ones. Prior to the analysis, data were Z-transformed to homogenise the variance. PCA analysis were performed using Statistica 13.0 software (TIBCO Software Inc., USA).

## Geographic coverage

### Description

Utrish State Nature Reserve, Abrau Peninsula, north-western Caucasus, Russia

### Coordinates

44.694123 N and 44.800702 N Latitude; 37.394033 E and 37.5495 E Longitude.

## Taxonomic coverage

### Description

All diatoms were identified to genus or species/intraspecific level. In total, 88 infrageneric taxa were identified belonging to two classes, 12 orders, 25 families and 39 genera distributed in the subphylum Bacillariophytina, 11 of them being identified only to genus level. The taxonomic coverage of the diatoms found in studied material is given in Table 2. The diatom species list with their ecological preferences, distribution and occurrence is given in Table 3.

## Temporal coverage

### Notes

July 12-20, 2022

## Usage licence

### Usage licence

Creative Commons Public Domain Waiver (CC-Zero)

## Data resources

### Data package title

Diatoms of Utrish State Nature Reserve, Abrau Peninsula (Russia)

### Resource link

https://www.gbif.org/occurrence/download?dataset_key=021f55ef-ec0c-427e-9ba5-bbfa0778bd64

### Alternative identifiers

http://gbif.ru:8080/ipt/resource?r=diatoms_utrish

### Number of data sets

1

### Data set 1.

#### Data set name

Diatoms of Utrish State Nature Reserve, Abrau Peninsula (Russia)

#### Data format

Darwin Core Archive

#### Data format version

1.1 published on 2022-06-20

#### Description

This dataset presents the first data on the distribution of freshwater and brackish diatoms on Abrau Peninsula and especially in the territory of the Utrish State Nature Reserve. The data in this occurrence resource have been published as a Darwin Core Archive (DwC-A), which is a standardised format for sharing biodiversity data as a set of one or more data tables. The core data table contains 215 occurrences. This IPT archives the data and, thus, serves as the data repository.

**Data set 1.** 

Column label	Column description
id	The ID of the record.
type	The nature of the resource.
basisOfRecord	The specific nature of the data record.
occurrenceID	Identifier of the record, coded as a global unique identifier.
eventID	Identifier of the event, unique for the dataset.
eventDate	Time interval when the event occurred.
country	Country of the sampling site.
countryCode	Code of the country where the event occurred.
LocationID	Identifier of sampling location for this dataset.
samplingProtocol	Description of sample collection method.
locationRemarks	Notes about the features of sampling site.
decimalLatitude	The geographic latitude of the sampling site.
decimalLongitude	The geographic longitude of the sampling site.
geodeticDatum	The spatial reference system upon which the geographic coordinates are based.
coordinateUncertaintyInMetres	The indicator for the accuracy of the coordinate location in metres, described as the radius of a circle around the stated point location.
recordedBy	A list (concatenated and separated) of names of people responsible for collecting material and recording the original Occurrence.
identifiedBy	A list (concatenated and separated) of names of people who assigned the Taxon to the subject.
taxonID	The identifier for the set of taxon information (data associated with the Taxon class). Specific identifier to the dataset.
scientificName	The name with authorship applied on the first identification of the specimen.
acceptedNameUsage	The specimen accepted name, with authorship.
kingdom	Kingdom name.
phylum	Phylum name.
class	Class name.
order	Order name.
family	Family name
genus	Genus name.
specificEpithet	The name of the first or species epithet of the scientificName.
infraspecificEpithet	The name of the lowest or terminal infraspecific epithet of the scientificName, excluding any rank designation.
taxonRank	The taxonomic rank of the most specific name in the scientificName.
scientificNameAuthorship	The authorship information for the scientificName.
identificationQualifier	Contains commentaries about taxon identification (marks sp., sensu lato etc.)

## Additional information

**Diatom diversity and occurrence**

This study presents 215 diatom (Bacillariophyta) occurrences in 67 sites on the Abrau Peninsula, belonging to 88 different infrageneric taxa from 39 genera, 25 families, 12 orders and one class (Table 2). Eleven of the 88 taxa have been identified only to genus level. No diatoms were found in 30 out of 67 samples. The families with the highest number of occurrences (> 10%) were Bacillariaceae (35; 16.3%), Gomphonemataceae (30; 14%), Naviculaceae (24; 11.2%), Achnanthidiaceae (24; 11.2%) and Catenulaceae (23; 10.7%). These families also were with the highest number of taxa: Bacillariaceae (13), Naviculaceae (8), Catenulaceae (7) and Gomphonemataceae (7), except for Achnanthidiaceae with three taxa. Additionally, the family Cymbellaceae was represented with a high number of taxa (7) despite the low occurrence rate (only 6.5%). The families with lower occurrences (< 3) were Bacillariophyceae *insertae sedis*, Neidiaceae, Pleurosigmataceae, Rhopalodiaceae, Staurosiraceae and Ulnariaceae (1; 0.5%) and Amphipleuraceae, Brachysiraceae, Fragilarialceae and Pinnulariaceae (2; 1%). All these families are families with the smallest number of diatom taxa: one in all, except for Pinnulariaceae with two taxa. The genera with the highest number of occurrences were *Gomphonema* (26), *Nitzschia* (22), *Navicula* (20), *Amphora* (14), *Cocconeis* (14), *Planothidium* (11) and *Achnanthes* (10). Thirty-six genera had less than five occurrences.

**Figure 2.**
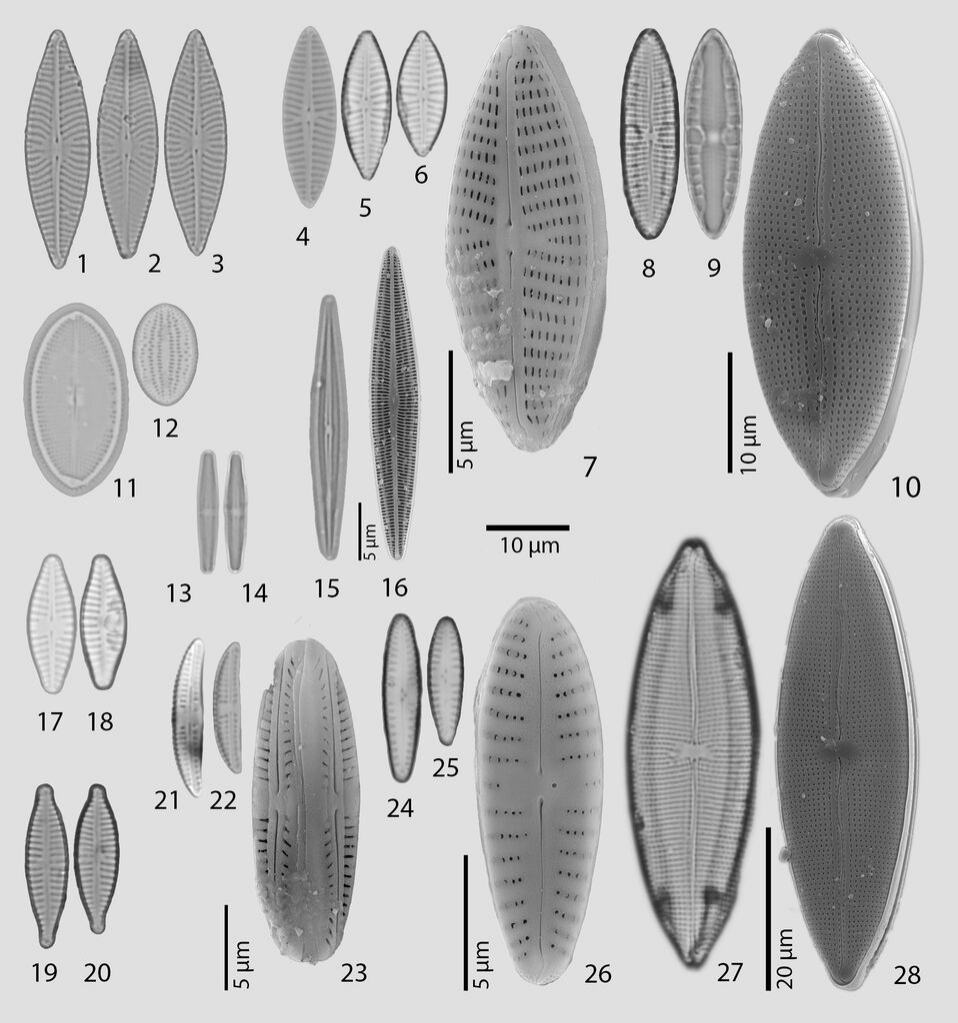
LM and SEM images of the most common and some other diatoms findings on Abrau Peninsula: **1-3** *Naviculablazencicae*; **4**-**7** – *Naviculaantonii*; **8-10** – *Mastogloia* sp.2; **11, 12** – *Cocconeisplacentula* s.l.; **13, 14** – *Achnanthidiumminutissimum*; **15, 16** – *Brachysiraaponina*; **17, 18** – *Planothidiumfrequentissimum*; **19, 20** – *Gomphonemaparvulum* s.l.; **21-23** – *Amphorainariensis*; **24-26** – Gomphonemapumilumvar.rigidum; **27, 28** – *Mastogloialanceolata*. Scale bar = 10 µm and applies for all images, except SEM pictures 7, 10, 16, 23, 26, 28. LM – light microscopy, SEM – scanning electron microscopy.

The most common species were *Planothidiumfrequentissimum* (11 samples), *Cocconeisplacentula* (9 samples), Gomphonemapumilumvar.rigidum (9 samples), *Achnanthidiumminutissimum* (8 samples), *Naviculaantonii* (7 samples), *Amphorainariensis* (6 samples) and *Gomphonemaparvulum* (6 samples) (Fig. 2).

The richiest sites in number of taxa were UT-2021-67 (20 taxa), UT-2021-20 (freshwater puddle, 16 taxa), UT-2021-28 (freshwater waterbody sediment, 14 taxa), UT-2021-25 (soil sample of puddle, 11 taxa), UT-2021-54 (freshwater waterbody with antropogenic impact, 11 taxa) and UT-2021-66 (coastline brackish lagoon, 11 taxa).

**Figure 3.**
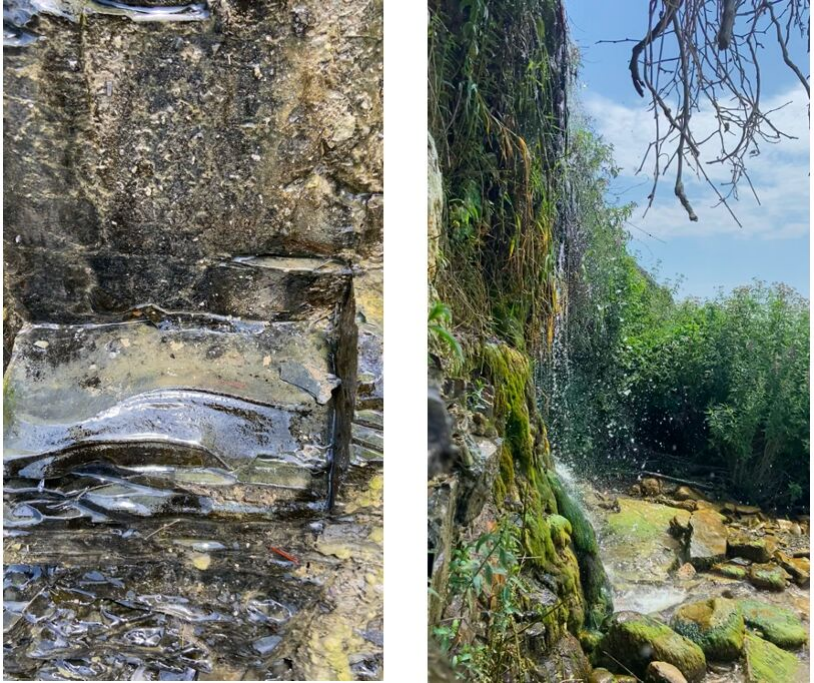
The view on Zhemchuzhnyj Waterfall, the hotspot of diatom diversity in Utrish Nature State Reserve. Rocks with algal film (left), stream water falls on stones covered with moss (right).

The UT-2021-67 site is a quite unique sampling site, where freshwater from the Zhemchuzhnyj Waterfall stream mixes with seawater and rocks with water from the stream being covered with moss. From this site, we sampled both rock scrap and moss squeeze and found the highest diversity of diatom taxa (Fig. 3).

Light microscope (LM) and scanning electron microscope (SEM) images of the most frequently occurring species and some others are represented in Fig. 2.

*Naviculablazencicae* Levkov (Fig. 2, 1-3) was originally described by Levkov and colleagues (Levkov et al. 2007) from North Macedonia and, until now, has been known as the endemic of Lake Ohrid. In the study, it was found in two sampling locations represented by two freshwater temporal waterbodies (UT-2021-05 and UT-2021-09).

One of *Mastogloia* species, referred to as *Mastogloia* sp.2 (Fig. 2, 8-10), held a unique combination of morphometric characteristics (paratecta and raphe structural features) which we were unable to identify as a known species. Probably the same species was also found by A. Kaleli (Kaleli 2019) in similar habitats of coastline brackish waterbodies. According to the published illustration (see *Mastogloia* sp.1 in Kaleli 2019), the valves collected by A. Kaleli are quite similar to *Mastogloia* sp.2 in the current study and supposedly belonged to the same species, although it has also not been identified and needs additional verification. Beside that, two other *Mastogloia* species (*Mastogloia* sp.1 and *Mastogloia* sp.3) which were found in the current research are also likely to be new species and require further study.

**Data analysis**

**Figure 4.**
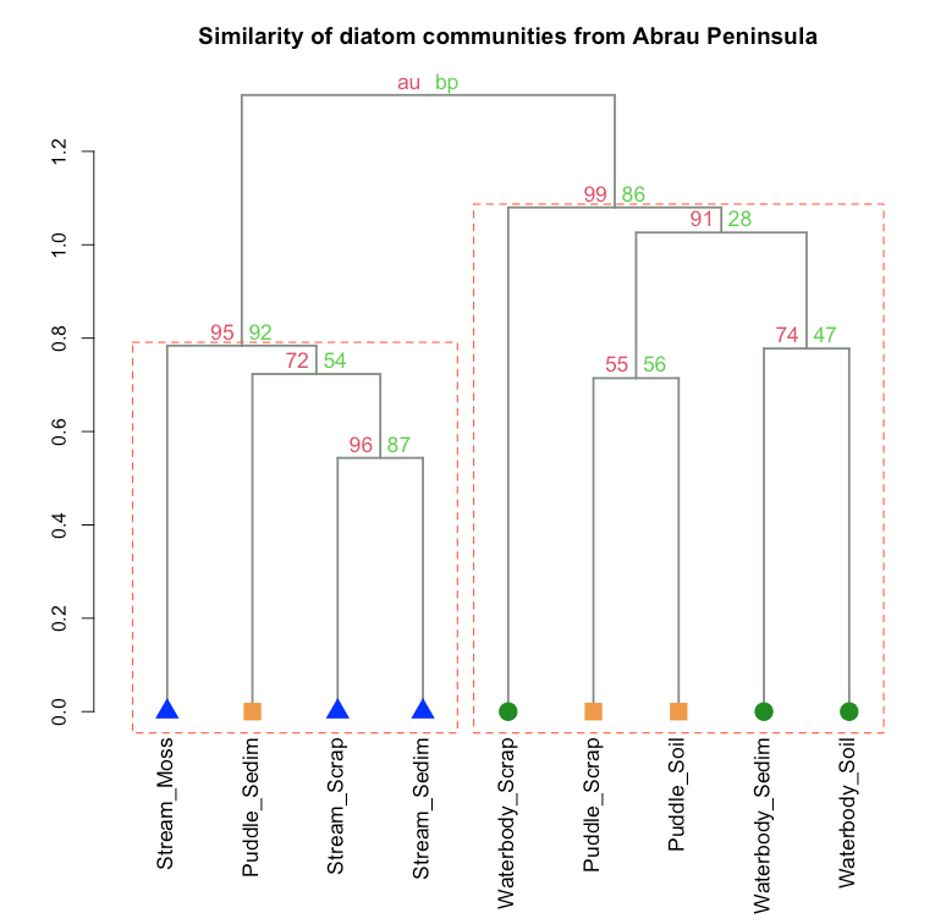
Hierarchical cluster analysis using the presence/absence matrix of diatom communities collected by different sampling methods from various biotopes of Abrau Peninsula (binary method, Ward.D2 clustering). Different symbols at the nodes of clusters illustrates biotope types: triangles – streams, squares – puddles (temporary waterbodies) and circles – permanent waterbodies. Right part of labels illustrates the type of sampling method: Moss – moss squeeze, Sedim – sediment from the bottom of waterbody or stream, Scrap – scrapping from the stones and Soil – soil in the littoral zone of waterbodies. Values at branches are approximately unbiased *p-values* (red colour) and bootstrap probabilities (green colour) in percentage. Clusters that are framed by red dashed line are supported by a *p-value* < 0.05.

The cluster analysis revealed a considerable modulation effect of habitat type on the floristic composition of diatom communities of the Abrau Peninsula (Fig. 4). Diatom communities collected from streams, regardless of the sampling method, were significantly dissimilar to the communities collected from waterbodies and communities collected from scraps and soils of puddles (p < 0.05). In turn, communities collected from waterbodies were combined with communities sampled from scraps and soils of puddles and formed significant clusters (p < 0.01).

The results of cluster analysis suggest that the floristic composition of diatom communities from streams is quite different from that in small ephemeral water objects (puddles) and stagnant water bodies (such as ponds, lakes and lagoons). Although some of the species living in streams might sometimes be present in puddles (see Fig. 4), the floristic composition of streams is most likely conservative and does not mix with other types of water objects.

**Figure 5.**
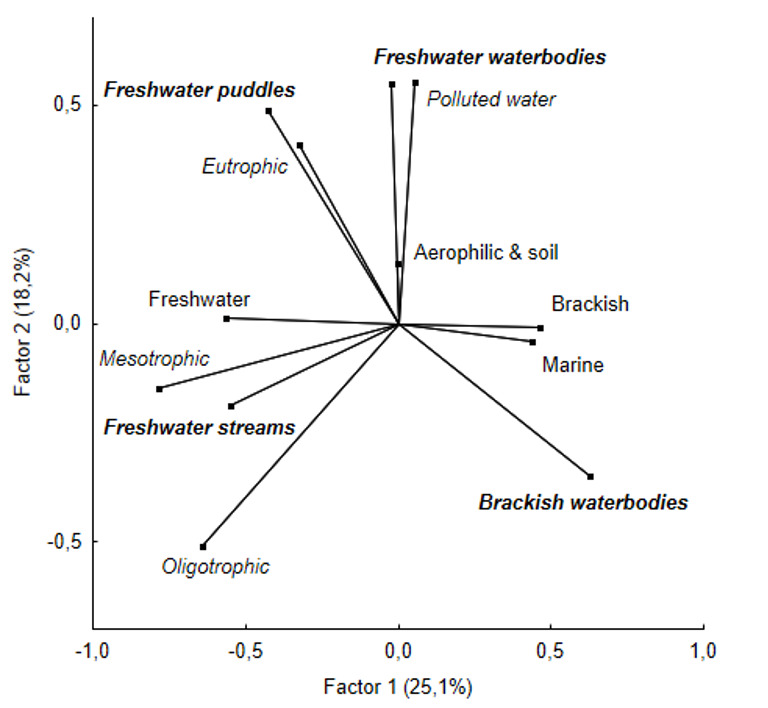
Relationship between frequency of species occurrences with different separability (italic, active variables) and environment preferences (normal, supplementary variables) and in various investigated habitats (bold and italic, active variables) determined using the principal component analysis (PCA).

The ecological conditions of marine and brackish waterbodies were obviously antagonistic to freshwater, thus the PCA by factor 1 clearly and predictably separated the frequency of freshwater and marine and brackish species (Fig. 5). The frequency of occurrence of oligotrophic and mesotrophic species strongly and positively correlated with freshwater streams of Abrau Peninsula. Only here were collected freshwater species such as *Amphorainariensis*, *A.pediculus*, *Cymbellaaffinis, C. hantzschiana*, *Encyonopsismicrocephala, Gomphonemapygmaeum, Naviculatripunctata* and *Reimeriauniseriata*. The majority of collected eutraphentic species tended to be from freshwater puddles (e.g. *Craticuladissociata*, *C.molestiformis*, *Gomphonemaparvulum*) and, to a lesser extent, freshwater waterbodies of the study area. The latter were positively correlated with the occurrence of species that prefer polluted water and, conversely, were antagonistic to the habitats of oligotrophic species and stream habitats. This may be due to the location of this type of waterbodies mainly near recreational areas and settlements. Aerophilic species did not show any strong correlation with the studied habitat types.

## Figures and Tables

**Table 1.** Samples code, location of the sampling sites, site type and sample type on the Abrau Peninsula.

Sampling code	Laboratory codename	Latitude (ºN)	Longitude (ºW)	Site type	Salinity	Sample type	Commentary
1	UT-2021-1	44.7582	37.4783	Stream	Freshwater	Rock scrap	No diatoms found
2	UT-2021-2	44.7582	37.4783	Stream	Freshwater	Rock scrap	
3	UT-2021-3	44.7637	37.4498	Temporary (Puddle)	Freshwater	Rock scrap	
4	UT-2021-4	44.7627	37.4556	Temporary (Puddle)	Freshwater	Sediment	No diatoms found
5	UT-2021-5	44.7623	37.4573	Temporary (Puddle)	Freshwater	Sediment	
6	UT-2021-6	44.7637	37.4497	Temporary (Puddle)	Freshwater	Soil	
7	UT-2021-7	44.7635	37.4520	Temporary (Puddle)	Freshwater	Soil	
8	UT-2021-8	44.7627	37.4556	Temporary (Puddle)	Freshwater	Sediment	No diatoms found
9	UT-2021-9	44.7627	37.4556	Temporary (Puddle)	Freshwater	Sediment	
10	UT-2021-10	44.7627	37.4556	Temporary (Puddle)	Freshwater	Sediment	No diatoms found
11	UT-2021-11	44.7582	37.4782	Stream	Freshwater	Rock scrap	No diatoms found
12	UT-2021-12	44.7582	37.4782	Stream	Freshwater	Rock scrap	No diatoms found
13	UT-2021-13	44.7635	37.4520	Temporary (Puddle)	Freshwater	Sediment	No diatoms found
14	UT-2021-14	44.7572	37.4713	Temporary (Puddle)	Freshwater	Rock scrap	No diatoms found
15	UT-2021-15	44.7606	37.4986	Temporary (Puddle)	Freshwater	Sediment	No diatoms found
16	UT-2021-16	44.7623	37.4573	Temporary (Puddle)	Freshwater	Sediment	No diatoms found
17	UT-2021-17	44.7097	37.4561	Permanent	Freshwater	Rock scrap	
18	UT-2021-18	44.7887	37.4765	Stream	Freshwater	Sediment	
19	UT-2021-19	44.7737	37.5125	Stream	Freshwater	Sediment	
20	UT-2021-20	44.7094	37.4562	Permanent	Freshwater	Sediment	
21	UT-2021-21	44.7239	37.4522	Temporary (Puddle)	Freshwater	Soil	No diatoms found
22	UT-2021-22	44.7887	37.4765	Stream	Freshwater	Rock scrap	No diatoms found
23	UT-2021-23	44.7151	37.4476	Permanent	Freshwater	Soil	
24	UT-2021-24	44.7552	37.4574	Temporary (Puddle)	Freshwater	Sediment	No diatoms found
25	UT-2021-25	44.7737	37.5125	Stream	Freshwater	Rock scrap	
26	UT-2021-26	44.7887	37.4764	Stream	Freshwater	Sediment	
27	UT-2021-27	44.7093	37.4561	Permanent	Freshwater	Sediment	
28	UT-2021-28	44.7178	37.5495	Stream	Freshwater	Sediment	
29	UT-2021-29	44.7851	37.4844	Stream	Freshwater	Sediment	
30	UT-2021-30	44.6946	37.5154	Stream	Freshwater	Rock scrap	
31	UT-2021-31	44.7606	37.4986	Temporary (Puddle)	Freshwater	Soil	
32	UT-2021-32	44.7888	37.4765	Stream	Freshwater	Rock scrap	
33	UT-2021-33	44.7851	37.4844	Stream	Freshwater	Rock scrap	
34	UT-2021-34	44.7737	37.5125	Stream	Freshwater	Rock scrap	
35	UT-2021-35	44.7888	37.4765	Stream	Freshwater	Rock scrap	No diatoms found
36	UT-2021-36	44.7247	37.4885	Stream	Freshwater	Rock scrap	No diatoms found
37	UT-2021-37	44.6941	37.5152	Stream	Freshwater	Rock scrap	
38	UT-2021-38	44.7247	37.4885	Stream	Freshwater	Rock scrap	No diatoms found
39	UT-2021-39	44.7178	37.5495	Stream	Freshwater	Rock scrap	
40	UT-2021-40	44.6945	37.5154	Stream	Freshwater	Rock scrap	No diatoms found
41	UT-2021-41	44.7178	37.5495	Stream	Freshwater	Rock scrap	No diatoms found
42	UT-2021-42	44.7888	37.4765	Stream	Freshwater	Rock scrap	No diatoms found
43	UT-2021-43	44.7093	37.4561	Permanent	Freshwater	Sediment	
44	UT-2021-44	44.7247	37.4885	Stream	Freshwater	Sediment	
45	UT-2021-45	44.7638	37.4498	Temporary (Puddle)	Freshwater	Sediment	No diatoms found
46	UT-2021-46	44.7582	37.4782	Stream	Freshwater	Sediment	No diatoms found
47	UT-2021-47	44.7247	37.4885	Stream	Freshwater	Rock scrap	No diatoms found
48	UT-2021-48	44.7247	37.4885	Stream	Freshwater	Rock scrap	No diatoms found
49	UT-2021-49	44.7709	37.5175	Temporary (Puddle)	Freshwater	Rock scrap	No diatoms found
50	UT-2021-50	44.7737	37.5125	Stream	Freshwater	Rock scrap	No diatoms found
51	UT-2021-51	44.7710	37.5156	Temporary (Puddle)	Freshwater	Sediment	No diatoms found
52	UT-2021-52	44.7690	37.5197	Stream	Freshwater	Rock scrap	No diatoms found
53	UT-2021-53	44.7697	37.5183	Temporary (Puddle)	Freshwater	Soil	No diatoms found
54	UT-2021-54	44.7690	37.5197	Stream	Freshwater	Rock scrap	
55	UT-2021-55	44.7862	37.4474	Temporary, Stream	Freshwater	Sediment	
56	UT-2021-56	44.7741	37.5111	Stream	Freshwater	Sediment	
57	UT-2021-57	44.7691	37.5199	Stream	Freshwater	Rock scrap	
58	UT-2021-58	44.7711	37.5155	Temporary (Puddle)	Freshwater	Sediment	No diatoms found
59	UT-2021-59	44.8007	37.4420	Stream	Freshwater	Rock scrap	
60	UT-2021-60	44.8007	37.4421	Stream	Freshwater	Rock scrap	No diatoms found
61	UT-2021-61	44.8007	37.4420	Stream	Freshwater	Rock scrap, Sediment	
62	UT-2021-62	44.7918	37.3940	Stream	Freshwater	Rock scrap	
63	UT-2021-63	44.7918	37.3940	Stream	Freshwater	Rock scrap, Sediment	
64	UT-2021-64	44.7254	37.4368	Stream	Freshwater	Rock scrap	
65	UT-2021-65	44.7345	37.4199	Permanent	Brackish	Rock scrap	
66	UT-2021-66	44.7354	37.4170	Permanent	Brackish	Rock scrap	
67	UT-2021-67	44.74781	37.4061	Stream	Brackish	Moss squeeze, Rock scrap	

**Table 2.** Taxonomic coverage of diatoms from studied samples.

Orders	Families	Genera	Total taxa	Total species
Achnanthales	3	4	10	8
Bacillariales	1	3	13	13
Bacillariophyta ordo incertae sedis	1	1	1	1
Cymbellales	2	7	14	12
Fragilariales	3	3	3	3
Licmophorales	1	1	1	
Mastogloiales	1	1	5	2
Naviculales	10	13	28	22
Rhabdonematales	1	2	2	2
Rhopalodiales	1	1	1	1
Surirellales	1	1	3	2
Thalassiophysales	1	2	7	7

**Table 3.** List of diatom species found in samples with notes on their ecology, distribution and occurrence (number of samples). Data on ecology and distribution are given according to Kulikovskiy et al. 2016, Cantonati et al. (2017), and Guiry and Guiry 2022 .

Taxa	Abbreviation for taxa	Habitat	Distribution	Saprobility	Water chemistry	Accuracy
Achnanthesbrevipesvar.brevipes C.Agardh	ACHBRE	Brackish, Marine	Widely distributed			1
*Achnanthes* sp.	ACHSP					1
*Achnanthidiumminutissimum* (Kützing) Czarnecki	ACHNMIN	Freshwater	Cosmopolitan			8
*Achnanthidium* sp.	ACHNSP					1
*Achnanthidiumstraubianum* (Lange-Bertalot) Lange-Bertalot	ACHNSTR	Freshwater	Arctic-alpine	Mesotrophic, Eutrophic	Calcium-bicarbonate rich	5
*Amphorainariensis* Krammer	AMINA	Freshwater	Widely distributed	Oligotrophic, Mesotrophic		4
*Amphoraindistincta* Levkov	AMINDI	Freshwater	Widely distributed	Oligotrophic		6
*Amphoraovalis* (Kützing) Kützing s.l.	AMOV	Freshwater	Cosmopolitan	Oligotrophic, Mesotrophic, Eutrophic		1
*Amphorapediculus* (Kützing) Grunow in A.W.F.Schmidt	AMPED	Freshwater, Brackish	Widely distributed	Oligotrophic		3
*Brachysiraaponina* Kützing	BRACH	Marine, Brackish	Widely distributed			3
Caloneiscf.vasileyevae Lange-Bertalot, Genkal & Vekhov	CALVAS	Freshwater	Holarctic			4
*Cocconeiseuglypta* Ehrenberg	COCCEU	Freshwater, Brackish	Cosmopolitan	Mesotrophic, Eutrophic	Alkaline	3
*Cocconeislineata* Ehrenberg	COCCLIN	Freshwater, Brackish	Cosmopolitan	Mesotrophic, Eutrophic	Alkaline	9
*Cocconeispediculus* Ehrenberg	COCCPED	Freshwater	Cosmopolitan	Mesotrophic, Eutrophic	Alkaline	1
*Cocconeisplacentula* Ehrenberg s.l.	COCCPLAT	Freshwater, Brackish	Cosmopolitan	Oligotrophic, Mesotrophic, Eutrophic		1
*Craticulaaccomoda* (Hustedt) D.G.Mann in Round, R.M.Crawford & D.G.Mann	CRATACC	Freshwater	Cosmopolitan	Eutrophic, Polluted water		1
Craticulacf.buderi (Grunov ex Van Heurck) D.G.Mann.	CRATBUD	Freshwater, Brackish	Widely distributed			1
*Craticuladissociata* (E.Reichardt) E.Reichardt	CRATDISS	Freshwater	Holarctic	Eutrophic		1
*Craticulamolestiformis* (Hustedt) Mayama	CRATMOL	Freshwater	Cosmopolitan	Eutrophic, Polluted water		1
*Ctenophora* sp.	CTENSP					1
*Cymbellaaffinis* Kützing	CYMAFF	Freshwater	Widely distributed, Alpine	Oligotrophic, Mesotrophic	Сalcium-bicarbonate rich	3
*Cymbellahantzschiana* Krammer	CYMHANTZ	Freshwater	Widely distributed	Oligotrophic, Mesotrophic		3
*Cymbopleura* sp.	CYMSP					1
*Diatomatenuis* C.Agardh	DIATTEN	Freshwater, Brackish	Cosmopolitan			2
Diploneiscf.carloswetzelii Lange-Bertalot & Fuhrmann	DIPCAR	Freshwater				1
*Diploneiskrammeri* Lange-Bertalot & E.Reichardt	DIPKRAM	Freshwater	Arctic-Alpine	Oligotrophic, Mesotrophic	Alkaline, Сalcium-bicarbonate rich	2
*Diploneisoculata* (Brébisson) Cleve	DIPOCU	Freshwater, Brackish	Cosmopolitan	Oligotrophic, Mesotrophic	Сalcium-bicarbonate rich	1
*Encyonopsismicrocephala* (Grunow) Krammer	ENCYMIC	Freshwater	Cosmopolitan	Oligotrophic, Mesotrophic	Сalcium-bicarbonate rich	4
*Encyonopsissubminuta* Krammer & E.Reichardt in Krammer	ENCYSUBM	Freshwater	Holarctic	Oligotrophic, Mesotrophic	Сalcium-bicarbonate rich	1
Fallaciacf.subhamulata (Grunow) D.G.Mann in Round, R.M.Crawford & D.G.Mann	ENCYSUBH	Freshwater	Holarctic	Oligotrophic, Mesotrophic	Alkaline	2
*Fragilariformabicapitata* (A.Mayer) D.M.Williams & Round	FRAGBIC	Freshwater	Holarctic	Oligotrophic, Mesotrophic, Eutrophic	Acidic, Siliceous	2
*Frustuliavulgaris* (Thwaites) De Toni	FRUSTV	Freshwater	Cosmopolitan	Mesotrophic, Eutrophic		2
*Geissleria* sp.	GEISSP	Freshwater				1
*Gomphonemaangustum* C.Agardh	GOMANG	Freshwater	Cosmopolitan		Calcium-bicarbonate rich	1
Gomphonemapumilumvar.rigidum E.Reichardt & Lange-Bertalot	GOMPUM	Freshwater	Cosmopolitan	Oligotrophic, Mesotrophic	Calcium-bicarbonate rich	9
*Gomphonemapygmaeum* J.Kociolek & E.Stoermer	GOMPYG	Freshwater	Holarctic			4
*Gomphonemamicropus* Kützing	GOMMIC	Freshwater, Brackish	Cosmopolitan	Oligotrophic, Mesotrophic	Alkaline	4
*Gomphonemaparvulum* (Kützing) Kützing s.l.	GOMPAR	Freshwater	Cosmopolitan	Mesotrophic, Eutrophic	Alkaline	6
*Gomphonemasubclavatum* (Grunow) Grunow	GOMSUB	Freshwater		Oligotrophic		2
*Halamphorabicapitata* (M.H.Hohn & J.Hellerman) J.G.Stepanek & Kociolek	HALABI		Holarctic			4
*Halamphoracoffeiformis* (C.Agardh) Mereschkowsky	HALACOFFE	Brackish	Cosmopolitan			1
*Halamphoramontana* (Krasske) Levkov	HALAMON	Freshwater	Cosmopolitan	Oligotrophic, Mesotrophic	Alkaline	4
*Hantzschiaamphioxys* (Ehrenberg) Grunow in Cleve & Grunow	HANTZAM	Freshwater	Cosmopolitan	Mesotrophic, Eutrophic		3
*Hantzschiaabundans* Lange-Bertalot	HANTZAB	Freshwater	Cosmopolitan	Mesotrophic, Eutrophic		1
*Humidophilacontenta* (Grunow) R.L.Lowe & al.	HUMCON	Freshwater, Aerophilic	Cosmopolitan			3
*Luticolaacidoclinata* Lange-Bertalot in Lange-Bertalot & Metzeltin	LUTAC	Freshwater, Aerophilic	Holarctic	Oligotrophic	Weakly acidic	1
Luticolacf.ventricosa (Kützing) D.G.Mann in Round, R.M.Crawford & D.G.Mann	LUTVEN	Freshwater, Aerophilic	Cosmopolitan			1
*Luticolamutica* (Kützing) D.G.Mann in Round, R.M.Crawford & D.G.Mann	LUTMUT	Freshwater, Brackish, Aerophilic	Cosmopolitan			2
*Luticolanivalis* (Ehrenberg) D.G.Mann in Round, R.M.Crawford & D.G.Mann	LUTNIV	Freshwater, Aerophilic	Holarctic	Oligotrophic		1
*Mastogloialanceolata* Thwaites ex W. Smith	MASTL	Brackish,Marine				2
Mastogloiapusillavar.pusilla Grunow	MASTP	Brackish, Marine				1
*Mastogloia* sp.1	MAST1	Brackish, Marine				1
*Mastogloia* sp.2	MAST2	Brackish, Marine				1
*Mastogloia* sp.3	MAST3	Brackish, Marine				2
Meridioncircularevar.constrictum (Ralfs) Van Heurck	MERCIR	Freshwater	Holarctic	Oligotrophic, Mesotrophic		2
*Naviculaantonii* Lange-Bertalot	NAVANT	Freshwater				7
*Naviculablazencicae* Z.Levkov & S.Krstic	NAVBLA	Freshwater	Alpine			2
*Naviculacincta* (Ehrenb.) Ralfs in A.Pritch.	NAVCINC					1
*Naviculacryptotenella*	NAVCRY					2
*Navicula* sp.	NAVSP					4
*Naviculatripunctata* (O.F.Müller) Bory in Bory de Saint-Vincent	NAVTRI	Freshwater	Cosmopolitan	Eutrophic		3
*Naviculavulpina* Kützing	NAVVUL	Freshwater	Cosmopolitan	Oligotrophic, Mesotrophic	Сalcium-bicarbonate rich	1
*Navicymbulapussila* (Grunow) Krammer	NAVYPUS	Brackish	Cosmopolitan		Сalcium-bicarbonate rich	1
*Neidiomorphabinodiformis* (Krammer) M.Cantonati, Lange-Bertalot & N.Angeli	NEIDBI	Freshwater	Holarctic	Oligotrophic		1
*Nitzschiaclausii* Hantzsch	NITZCLAUS	Freshwater, Brackish	Cosmopolitan	Mesotrophic		1
*Nitzschiadenticula* Grunow	NITZDEN	Freshwater	Widely distributed	Oligotrophic, Mesotrophic	Сalcium-bicarbonate rich	4
*Nitzschialinearis* W.Smith	NITZLIN	Freshwater	Holarctic	Eutrophic	Alkaline	4
*Nitzschiaschwabei* Krasske ex Lange-Bertalot	NITZSCH	Brackish	Holarctic			4
*Nitzschiatenuis* W.Smith	NITZTE	Freshwater	Holarctic	Eutrophic		1
*Nitzschiathermaloides* Hustedt	NITZTHE	Marine, Brackish	Holarctic			2
*Nitzschiatubicola* Grunow in Cleve & Grunow	NITZTU	Marine, Brackish	Cosmopolitan			5
*Nitzschiavaldestriata* Aleem & Hustedt	NITZVA	Freshwater, Brackish	Widely distributed			1
Pinnulariabertrandiivar.angustefasciata Krammer	PINNBET	Freshwater	Holarctic			1
Pinnulariaborealisvar.scalaris (Ehrenberg) Rabenhorst	PINNBOR	Freshwater	Widely distributed		Siliceous	1
*Planothidiumfrequentissimum* (Lange-Bertalot) Lange-Bertalot	PLANFRE	Freshwater	Cosmopolitan	Oligotrophic, Mesotrophic	Alkaline	11
*Playaensiscitrus* (Krasske) E.Reichardt	PLAYCI	Freshwater	Widely distributed			1
*Pleurosigmaelongatum* W.Smith	PLEU					1
*Pseudostaurosirabrevistriata* (Grunow) D.M.Williams & Round	PSEUSBRE	Freshwater, Brackish	Cosmopolitan	Oligotrophic, Mesotrophic, Eutrophic	Calcium-bicarbonate rich	1
*Reimeriauniseriata* S.E.Sala, J.M.Guerrero & M.E.Ferrario	REIMUN	Freshwater	Widely distributed			4
*Rhopalodiagibba* (Ehrenberg) O.Müller	RHOGI	Freshwater	Cosmopolitan	Oligotrophic, Mesotrophic, Eutrophic	Alkaline	1
*Sellaphora* sp.	SELLSP					1
*Stauroformaexiguiformis* (Lange-Bertalot) R.J.Flower, V.J.Jones & Round	STAUREXI	Freshwater	Cosmopolitan	Eutrophic	Acidic	1
*Surirellaangusta* Kützing	SURAN	Freshwater	Widely distributed	Mesotrophic, Eutrophic		1
*Surirellaovalis* Brébisson	SUROV	Brackish, Marine	Cosmopolitan			1
*Surirella* sp.	SURSP	Brackish, Marine				1
*Tryblionellaangustata* W.Smith	TRYAN	Freshwater, Brackish, Marine	Cosmopolitan			2
*Tryblionellaapiculata* W.Gregory	TRYAP	Freshwater, Brackish, Marine	Cosmopolitan	Oligotrophic, Mesotrophic		2
*Tryblionellahungarica* (Grunow) Frenguelli	TRYHUN	Brackish	Cosmopolitan	Mesotrophic		5
